# Joint Sensor Selection and Power Allocation Algorithm for Multiple-Target Tracking of Unmanned Cluster Based on Fuzzy Logic Reasoning

**DOI:** 10.3390/s20051371

**Published:** 2020-03-02

**Authors:** Yuanshi Zhang, Minghai Pan, Qinghua Han

**Affiliations:** College of Electronic and Information Engineering, Nanjing University of Aeronautics and Astronautics, Nanjing 211106, China; Z_yuanshi@163.com (Y.Z.); panmh@nuaa.edu.cn (M.P.)

**Keywords:** radar resource management, radar networks, unmanned aerial vehicle cluster, chance-constraint programming (CCP), fuzzy logic reasoning (FLR)

## Abstract

The unmanned aerial vehicle (UAV) cluster is gradually attracting more attention, which takes advantage over a traditional single manned platform. Because the size of the UAV platform limits the transmitting power of its own radar, how to reduce the transmitting power while meeting the detection accuracy is necessary. Aim at multiple-target tracking (MTT), a joint radar node selection and power allocation algorithm for radar networks is proposed. The algorithm first uses fuzzy logic reasoning (FLR) to obtain the priority of targets to radars, and designs a radar clustering algorithm based on the priority to form several subradar networks. The radar clustering algorithm simplifies the problem of multiple-radar tracking multiple-target into several problems of multiple-radar tracking a single target, which avoids complex calculations caused by multiple variables in the objective function of joint radar node selection and power allocation model. Considering the uncertainty of the target RCS in practice, the chance-constraint programming (CCP) is used to balance power resource and tracking accuracy. Through the joint radar node selection and power allocation algorithm, the radar networks can use less power resource to achieve a given tracking performance, which is more suitable for working on drone platforms. Finally, the simulation proves the effectiveness of the algorithm.

## 1. Introduction

Unmanned cluster has strong battlefield viability, and radar networks based on unmanned clusters have received much attention. Compared with traditional monostatic radar, radar networks based on clustered drones have a high degree of freedom in array layout and can be adjusted according to the battlefield environment flexibly [1,2].

The resource management of the radar networks includes the management of the composition of the radar networks and the allocation of the radar transmit resource [3]. For the management of radar networks component structure, Godrich solved the problem of radar node selection by greedy algorithm under the constraints of target detection accuracy [4]. He, Q obtained the optimal radar node deployment scheme for a given detection performance by using the Cramer–Rao lower bound (CRLB) of the target speed estimation as the objective function [5]. In view of the management of radar transmit resource, Ye proposed a scaled accuracy-based power allocation framework for multiple-target tracking (MTT), achieves the effective overall performance of MTT while taking into account the differences targets priorities [6]. According to the characteristics of the target and the battlefield, Shi proposed a scheme to meet the target tracking performance by adjusting the transmit power and dwelling strategy, which also can achieve good radio frequency (RF) stealth performance [7]. Yan developed a collaborative detection and power allocation scheme, which can evidently expand the detection range, increase the resource utilization efficiency of the multiple-radar system, and improve the target tracking accuracy [8]. However, the above literature only considers the management of a single radar resource that cannot adapt to complex radar networks.

The layout structure of the radar networks is spatially dispersed. So, the resource management of radar networks can jointly allocate the radar node and transmit resources. Aiming at multiple-radar tracking multiple targets, Yan proposed a radar clustering and power allocation algorithm that can select a specific number of radars to cluster tracking each target, and optimize radar transmit power on the basis of tracking accuracy [9]. Andargoli studied the multi-base radar system in multi-beam operation mode; the algorithm reduces the tracking error by the joint radar beam and transmit power management [10]. A joint radar node selection and power allocation model for distributed radar is built in reference [11], which is constrained by tracking accuracy of target. The problem was solved by transforming it into a second-order cone programming. Chavali studied the radar node selection and power allocation of cognitive radar systems under tracking tasks by establishing a specified transmission model and solves this problem with a greedy algorithm [12]. Using mutual information and minimum mean square error as the measurement index of target tracking performance, She established a transmission power optimization model based on RF stealth for airborne networking radar multiple-target tracking and uses two-step method to solve it [13]. Yu et al. proposed a Compressed Sensing (CS)-based distributed MIMO radar system power allocation strategy. The purpose of this strategy is to minimize the correlation between target echoes from different search units. Compared with the equal power allocation strategy, this strategy can greatly improve target detection performance [14]. Xie et al. used optimal fusion technology to build a joint node selection and power allocation model under the decentralized topology of the radar networks and solved by a two-step method [15].

The above papers lay a solid foundation for the research of radar networks resource management and provide many ideas for follow-up researchers, but there are several shortcomings: (1) The above research consider the radar cross section (RCS) of the target as a deterministic variable. Due to the changeable environment and unknown target information, the RCS of the target is uncertain. Under uncertain conditions, it is difficult to guarantee the robustness of the algorithm by using a deterministic model to allocate resources [16]. (2) In the above-mentioned scheme for joint radar node and transmit parameters management, the objective function always contains multiple variables, which often requires algorithms with a large amount of calculations to solve. 

Aiming at the above problems, this paper proposes radar node selection and power allocation chance-constraint programming(CCP) algorithm based on fuzzy logic reasoning(FLR) in the case of multiple-target tracking. At each moment, the algorithm first uses FLR to obtain the priority of each target to the radar. Then fusion center clusters the radars and the targets according to the priority to split the multiple-radar tracking multiple-target system into several subsystems of multiple-radar tracking single target, which can avoid the occurrence of two variables—radar node and transmit power—in the optimization function. For the management of the transmit power, a transmit power CCP model is constructed. The CCP model can not only deal with the uncertainty of target measurement, but also ensure the robustness of the algorithm [17]. This algorithm can intelligently schedule multiple radars to track appropriate targets and optimize the transmission power of each radar at the same time, making the radar networks system more suitable for miniaturized unmanned aerial vehicle(UAV) platforms.

The remaining structure of the article is as follows. The system model is constructed in the Section 2. In Section 3, the principle of FLR is introduced, and a radar-clustering algorithm based on FLR is designed, which is also a major innovation of this paper. In Section 4, based on the completion of radar clustering, a transmit power CCP model for multiple-radar tracking multiple-target is constructed, and it is split into several transmit power CCP model for a multiple-radar tracking single target. Section 5 introduces the target state estimate algorithm. Section 6 presents the simulation results. Finally, the conclusion is given in Section 7.

## 2. System Structure

**Figure 1:**
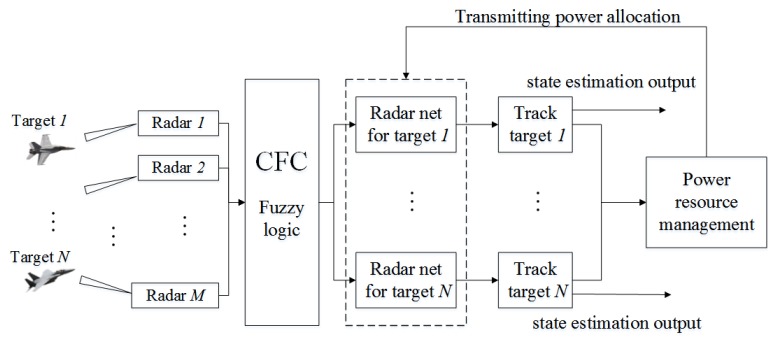
Radar network resource management process.

Assume a scene with *M* radars tracking *N* targets. Each radar only tracks one target at a time. In every sampling period, each radar sends the target information obtained from the echo signal to the fusion center. The fusion center uses the information to guide the operation of the radar system at next moment. The flow chart of the system is shown in Figure 1.

### 2.1. Target Motion Model

Assume that all targets move in a straight line at a constant speed, the expression is as follows:

(1) $$\xi_{q,k}=F\xi_{q,k-1}+u_{q,k-1}$$

where $\xi_{q,k}={\left[x_{k},{\dot{x}}_{k},y_{k},{\dot{y}}_{k}\right]}^{T}$ denotes the state vector of the target *q* at the *k*th sample interval. ${\left[x_{k},y_{k}\right]}^{T}$ and ${\left[{\dot{x}}_{k},{\dot{y}}_{k}\right]}^{T}$ represents position and velocity vector of the target, respectively. F is a 4 × 4 state transition matrix:

(2) $$F=I_{2}⊗\left[\begin{array}{cc} 1 & T_{k}\\ 0 & 1 \end{array}\right]$$

where $I_{2}$ is second-order identity matrix. $T_{k}$ is the tracking sample interval, ⊗ represents the direct product of the matrix. $u_{q,k-1}$ denotes zero mean Gaussian white noise, and its covariance is:

(3) $$Q_{1,k-1}=\gamma_{1}\cdot I_{2}⊗\left[\begin{array}{cc} \frac{1}{3}T_{k}^{3} & \frac{1}{2}T_{k}^{2}\\ \frac{1}{2}T_{k}^{2} & T_{k} \end{array}\right]$$

where $\gamma_{1}$ is the noise intensity [18].

### 2.2. Measurement Model

Radar obtains target range, azimuth and Doppler information from target echoes. At the *k*th sample interval, the nonlinear measurement equation of the target *q* observed by the radar *i* is:

(4) $$z_{q,k}^{i}=h\left(\xi_{q,k}\right)+w_{q,k}^{i}$$

where

(5) $$h\left(\xi_{q,k}\right)={\left(R_{q,k}^{i},\theta_{q,k}^{i},f_{q,k}^{i}\right)}^{T}$$

$R_{q,k}^{i}$, $\theta_{q,k}^{i}$ and $f_{q,k}^{i}$ are distance, azimuth and Doppler frequency information respectively:

(6) $$\{\begin{array}{l} R_{q,k}^{i}=\sqrt{{\left(x_{q,k}-x_{i}\right)}^{2}+{\left(y_{q,k}-y_{i}\right)}^{2}}\\ \theta_{q,k}^{i}=\arctan\left[\left(y_{q,k}-y_{i}\right)/\left(x_{q,k}-x_{i}\right)\right]\\ f_{q,k}^{i}=-\frac{2}{\lambda_{q}}\left(x_{q,k},y_{q,k}\right)\times {\left(x_{q,k}-x_{i},y_{q,k}-y_{i}\right)}^{T}/R_{q,k}^{i} \end{array}$$

where $\lambda_{q}$ represents carrier wavelength of radar *q*. $w_{q,k}^{i}$ denotes zero-mean Gaussian white noise, and its covariance is:

(7) $$\sum_{q,k}^{i}=blkdiag\left(\sigma_{R_{q,k}^{i}}^{2},\sigma_{\theta_{q,k}^{i}}^{2},\sigma_{f_{q,k}^{i}}^{2}\right)$$

Where $\sigma_{R_{q,k}^{i}}^{2}$, $\sigma_{\theta_{q,k}^{i}}^{2}$ and $\sigma_{f_{q,k}^{i}}^{2}$ represent the measurement variances of distance, azimuth, and Doppler frequency respectively [19], and their expressions are:

(8) $$\{\begin{array}{l} \sigma_{R_{q,k}^{i}}^{2}=c^{2}/\left[32\pi^{2}\times {\mathrm{SNR}}_{q,k}^{i}\times {\left(B_{q,k}^{i}\right)}^{2}\right]\\ \sigma_{\theta_{q,k}^{i}}^{2}=3{\left(B_{NN}\right)}^{2}/\left(8\pi^{2}\times {\mathrm{SNR}}_{q,k}^{i}\right)\\ \sigma_{f_{q,k}^{i}}^{2}=3/\left[8\pi^{2}\times {\mathrm{SNR}}_{q,k}^{i}\times {\left(T_{q,k}^{i}\right)}^{2}\right] \end{array}$$

where $B_{q,k}^{i}$ and $T_{q,k}^{i}$ represent limited bandwidth and time width respectively.$B_{NN}$ represents the zero power beam width of the receiving antenna. *c* is speed of light. Assuming that there is no deviation between the radar beam irradiation direction and the azimuth of the target *q*, the expression of the signal-to-noise ratio of the echo of the target *q* received by the radar *i* is [16]:

(9) $${\mathrm{SNR}}_{q,k}^{i}\propto \alpha_{q,k}^{i}\left|h_{q,k}\right|P_{q,k}^{i}\Leftrightarrow \frac{{\left|h_{q,k}\right|}^{2}P_{q,k}^{i}}{{\left(R_{q,k}^{i}\right)}^{{}^{4}}}$$

where $R_{q,k}^{i}$ represents transmit power of the radar *i*. $\alpha_{q,k}^{i}\propto 1/{\left(R_{q,k}^{i}\right)}^{4}$ represents transmit power of the radar *i*.represents signal strength attenuation along the transmission path.

### 2.3. Fusion Center

Clustered radars send the measurement information to the fusion center for further processing. At the *k*th sample interval, the measurement of target *q* can be obtained from the fusion center:

(10) $$Z_{q,k}={\left[{\left(R_{q,k}\right)}^{T},{\left(\theta_{q,k}\right)}^{T},{\left(f_{q,k}\right)}^{T}\right]}^{T}+{\left[{\left(\Delta R_{q,k}\right)}^{T},{\left(\Delta \theta_{q,k}\right)}^{T},{\left(\Delta f_{q,k}\right)}^{T}\right]}^{T}R_{q,k}=\left[R_{q,k}^{1},R_{q,k}^{2},\ldots ,R_{q,k}^{m}\right]$$

where $R_{q,k}=\left[R_{q,k}^{1},R_{q,k}^{2},\ldots ,R_{q,k}^{m}\right]$, $\theta_{q,k}=\left[\theta_{q,k}^{1},\theta_{q,k}^{2},\ldots ,\theta_{q,k}^{m}\right]$ and $f_{q,k}=\left[f_{q,k}^{1},f_{q,k}^{2},\ldots ,f_{q,k}^{m}\right]$ represent the measurement set of the distance, azimuth, and Doppler frequency information of target *q* at the *k*th sample interval respectively.$\Delta R_{q,k}=\left[\Delta R_{q,k}^{1},\Delta R_{q,k}^{2},\ldots ,\Delta R_{q,k}^{m}\right]$, $\Delta \theta_{q,k}=\left[\Delta \theta_{q,k}^{1},\Delta \theta_{q,k}^{2},\ldots ,\Delta \theta_{q,k}^{m}\right]$ and $\Delta f_{q,k}=\left[\Delta f_{q,k}^{1},\Delta f_{q,k}^{2},\ldots ,\Delta f_{q,k}^{m}\right]$ represent the noise vectors of the radar measurement parameters. The measurement errors of each radar are independent, and the covariance matrix of the measurement noise vector can be expressed as:

(11) $$G_{q,k}=diag\left\{\sigma_{R_{q,k}^{1}}^{2},\sigma_{R_{q,k}^{2}}^{2},\ldots ,\sigma_{R_{q,k}^{m}}^{2},\sigma_{\theta_{q,k}^{1}}^{2},\sigma_{\theta_{q,k}^{2}}^{2},\ldots ,\sigma_{\theta_{q,k}^{m}}^{2},\sigma_{f_{q,k}^{1}}^{2},\sigma_{f_{q,k}^{2}}^{2},\ldots ,\sigma_{f_{q,k}^{m}}^{2}\right\}$$

where diag{·} represents diagonal matrix.

## 3. Radar Cluster

**Figure 2:**
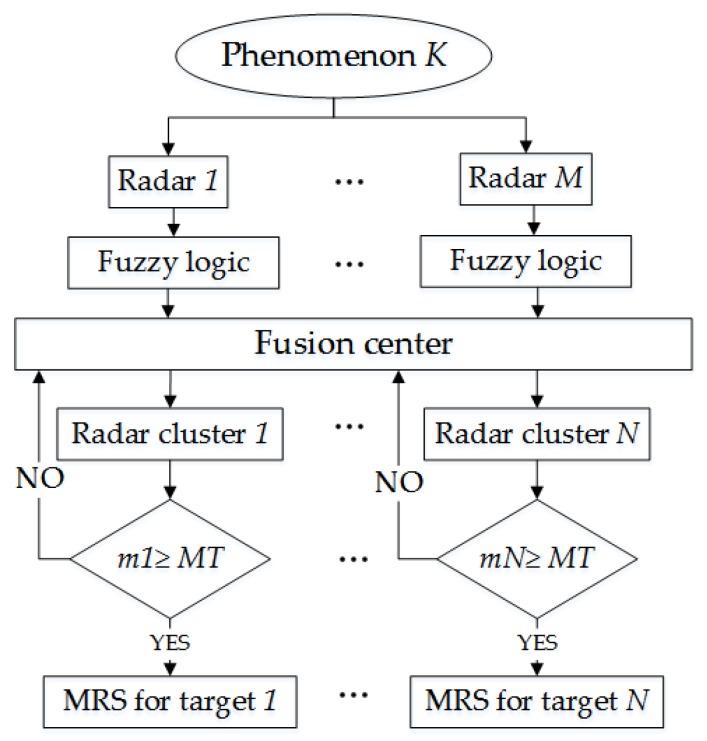
Flow chart of radar clustering.

The radar networks based on unmanned cluster can adjust the form of radar networking according to the battlefield environment. This paper uses FLR to reasonably cluster radars at every moment. To ensure that each target is always observed by a fixed number of radars, the algorithm is designed as shown in Figure 2:

Where *MT* is the preset number of radars that track each target.

### 3.1. Target Priority Based on Fuzzy Logic Reasoning

**Figure 3:**
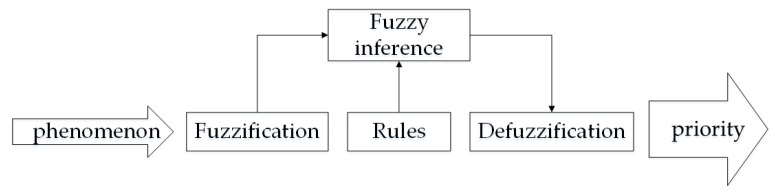
Flow chart of fuzzy logic reasoning (FLR) system.

FLR is a method for uncertain reasoning problem. It is based on fuzzy logic and uses information such as target attributes and the knowledge base in view of the intuitive and expert considerations to calculate the priority of a target online. The FLR system is shown in Figure 3 [20]:

#### 3.1.1. Fuzzification

Fuzzification is essentially a concept of map introduced in mathematics, which describes the relationship between the exact numerical value and the fuzzy sets [21]. This paper uses triangular fuzzifier to fuzzify the input numerical value. Its expression is as follows:

(12) $$\mu_{A^{\prime }}\left(x\right)=\{\begin{array}{cc} \left(1-\frac{\left|x-x^{*}\right|}{\sigma }\right) & if\left|x-x^{*}\right|\leq \sigma \\ 0 & otherwise \end{array}$$

where *σ* is a positive number, whose size is proportional to the anti-interference ability of the triangle fuzzy method. Here we consider three factors that are important to the target as input variables for fuzzy logic reasoning: (1) distance; (2) speed; (3) target identity. The detailed description of these three variables is shown in the Table 1 [22,23].

#### 3.1.2. Fuzzy Rule

The fuzzy rule base provides control rules for fuzzy reasoning, which is related to the distribution of fuzzy values. The more fuzzy the values, the more rules. The form of the fuzzy logic rules in this paper could be represented as:

(13) $$R_{i}:\mathrm{if}\;x_{1}\;\mathrm{is}\;A_{i}\;\mathrm{AND}\;x_{2}\;\mathrm{is}\;B_{i}\;\mathrm{AND}\;x_{3}\;\mathrm{is}\;C_{i}\;\mathrm{then}\;y\mathrm{is}\;D_{i}$$

Based on intuitive and expert considerations, 75 if-then fuzzy rules are obtained, as shown in the Appendix A.

#### 3.1.3. Fuzzy Inference

Firstly, the fuzzy inference needs to match the appropriate rules for the current input value, then use the fuzzy rules to operate on the input value, and finally get the fuzzy output value. According to the Mamdani fuzzy inference method, the fuzzy relation matrix $\mu_{R_{i}}$ of the *i*th fuzzy rule can be obtained [24].

(14) $$\mu_{R_{i}}\left(x,y,z,w\right)=\left[\mu_{A_{i}}\left(x\right)\wedge \mu_{B_{i}}\left(x\right)\wedge \mu_{C_{i}}\left(x\right)\right]\wedge \mu_{D_{i}}\left(x\right)$$

where μ is membership function, ∧ is the minimum operator and x∈X,y∈Y,z∈Z,w∈W.

The fuzzy relation *R* constituted by all fuzzy rules *R_i_* is:

(15) $$R=\underset{i=1,2,\ldots ,N}{\cup }R_{i}$$

where ∪ is the fuzzy union operation, so the conclusion *D’* is:

(16) $$\begin{array}{ll} D^{\prime } & =\left(A^{\prime }\mathrm{AND}B^{\prime }\mathrm{AND}C^{\prime }\right)\cap R\\ & =\left(A^{\prime }\mathrm{AND}B^{\prime }\mathrm{AND}C^{\prime }\right)\cap \left(\underset{i=1,2,\ldots ,N}{\cup }R_{i}\right)\\ & =D_{1}^{\prime }\cup D_{2}^{\prime }\cup \ldots D_{N}^{\prime } \end{array}$$

where ∩ is the fuzzy intersection operation, and $D_{i}^{\prime }$ is:

(17) $$D^{\prime }=\left(A^{\prime }\mathrm{AND}B^{\prime }\mathrm{AND}C^{\prime }\right)\cap \left[\left(A_{i}\mathrm{AND}B_{i}\mathrm{AND}C_{i}\right)\to D_{i}\right]$$

So, the membership function of $D_{i}^{\prime }$ is:

(18) $$\begin{array}{ll} \mu_{D_{i}^{\prime }}\left(w\right) & =\left[\mu_{A_{i}^{\prime }}\left(x\right)\wedge \mu_{A_{i}}\left(x\right)\right]\wedge \left[\mu_{A_{i}^{\prime }}\left(y\right)\wedge \mu_{A_{i}}\left(y\right)\right]\wedge \left[\mu_{A_{i}^{\prime }}\left(z\right)\wedge \mu_{A_{i}}\left(z\right)\right]\wedge \mu_{D_{i}}\left(w\right)\\ & =\gamma_{i}\wedge \mu_{D_{i}}\left(w\right) \end{array}$$

where $\gamma_{i}$ is the height of the intersection of the $A_{i}$, $B_{i}$, $C_{i}$ and the intersection of, $A^{\prime }$, $B^{\prime }$ and $C^{\prime }$, which can be understood as fitness of the $A_{i}$,$B_{i}$, $C_{i}$ and the intersection of $A^{\prime }$, $B^{\prime }$ and $C^{\prime }$.

**Figure 4:**
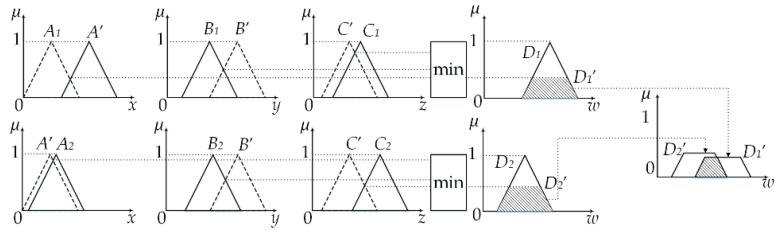
Three-input two-rule reasoning.

The geometric property of fuzzy inference is to use $\gamma_{i}$ as the reference cutting set $D_{i}$, and then get the conclusion *D’*. Figure 4 shows the geometric properties of multiple condition fuzzy inference when the number of rules is 2.

#### 3.1.4. Defuzzification

The results obtained by fuzzy inference are fuzzy, which cannot be used to directly measure the priority of the target. So, the fuzzy value need to convert to the exact numerical value. Defuzzification can be regarded as the mapping of fuzzy space to exact numerical space. This paper uses the center average defuzzifier for defuzzification. In this method, after weighted average, the output numerical value of each element is taken as the execution quantity. Where, the weight is the height of the corresponding fuzzy set [25]. The numerical value calculated by the following formula:

(19) $$w^{*}=\frac{\sum_{i=1}^{N}\left(w_{i}^{*}\times \mu_{max}^{i}\left(w\right)\right)}{\sum_{i=1}^{N}\mu_{max}^{i}\left(w\right)}$$

where $w_{i}^{*}$ is the center of mass of the *i*th fuzzy set, $\mu_{max}^{i}\left(w\right)$ is the maximum membership function of the fuzzy set. $w^{*}$ is the numerical result calculated by center average defuzzifier, that is, the target priority calculated by the entire FLR.

### 3.2. Radar Clustering Strategy Based on the Target Priority

The priority of each target for different radar at the *k*th sample interval is constituted into M×N matrix $W_{k}$(*M* is the number of radar, *N* is the number of target), then the radars are clustered by the following methods.

**Figure 5:**
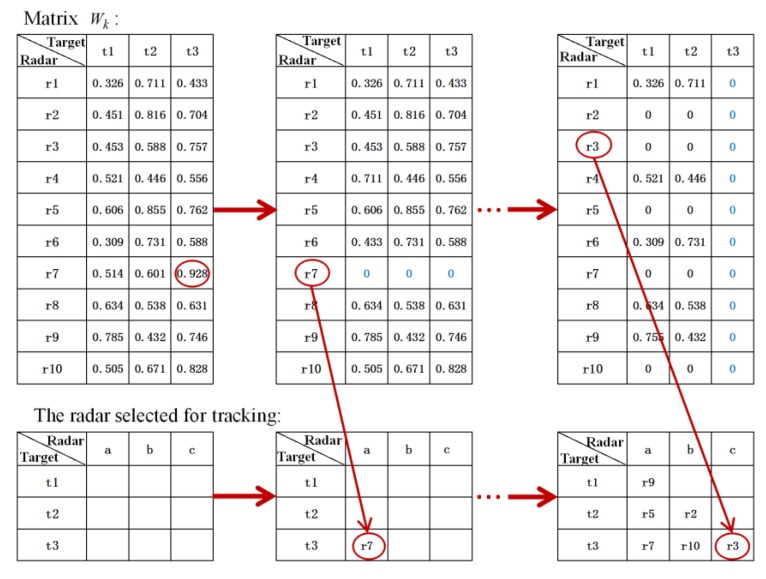
Example of radar assignment process.

1. Select the highest priority value $w_{n_{k}}^{m_{k}}$ from the matrix $W_{k}$. Then the radar *m_k_* corresponding to $w_{n_{k}}^{m_{k}}$ is assigned to the radar cluster that will track the target *n_k_*. Set all values of the matrix $W_{k}$ in line *m_k_* to zeros.
2. Repeat step 1 to assign radars to each target. If the number of radars assigned to a target has reached the preset require number *MT*, set all values in the column of the target in matrix $W_{k}$ to 0. Figure 5 shows the flow of radar clustering algorithm.

By using the above radar-clustering algorithm based on FLR at each sample interval, each radar can track the target with high priority to itself. 

## 4. Management of Power Resource of Radar Networks

In order to deal with the uncertainty of the environment and target information, this paper treats target RCS as uncertainty information. So, the power resource CCP model for multiple-radar tracking multiple-target is constructed. The Bayesian Cramer-Rao low bound (BCRLB) is used to measure the tracking accuracy of the targets. With the radar-clustering algorithm based on FLR proposed in the previous section, the multiple-radar tracking multiple-target system is divided into *N* (the number of target) multiple-radar tracking single-target subsystems. Then, aiming at the subsystem, the transmitting power CCP submodel for multiple-radar tracking single-target is established. The submodel is solved by the hybrid intelligence algorithm, which is formed by embedding the stochastic simulation into the genetic algorithm.

### 4.1. BCRLB of Target Tracking

In Bayesian estimation, for the target of a single motion model, the BCRLB matrix provides a lower bound for the MSE of the state estimation of the target [26]:

(20) $${Ε}_{\xi_{q,k},Z_{q,k}}\left[\left({\hat{\xi }}_{q,k}\left(Z_{q,k}\right)-\xi_{q,k}\right){\left({\hat{\xi }}_{q,k}\left(Z_{q,k}\right)-\xi_{q,k}\right)}^{T}\right]\geq C_{BCRLB}^{q,k}\left(\xi_{k}\right)=J^{-1}\left(\xi_{q,k}\right)$$

where, $J^{-1}\left(\xi_{q,k}\right)$ represents the inverse of the BIM matrix, $E_{\xi_{q,k},Z_{q,k}}\left[\cdot \right]$ is the expected value of $Z_{q,k}$.${\hat{\xi }}_{q,k}$ is the predicted value of the state vector $\xi_{q,k}$ of the target *q* [27]. $Z_{q,k}={\left\{z_{k}^{i}\right\}}_{i=1}^{m}$ is the measurement set of the radar observing the target *q*. *m* is the number of radars clustered to observe the target *q* in the same times.

At the *k*th sample interval, there are *m* radars for clustering observation of the target *q*, so the BIM matrix $J\left(\xi_{q,k}\right)$ of the target *q* is [26]:

(21) $$J\left(\xi_{q,k}\right)={\left[Q_{q,k-1}+F_{q,k}J^{-1}\left(\xi_{q,k}\right)F_{q,k}{}^{T}\right]}^{-1}+{\left(\sum_{i=1}^{m}{\left(H_{q,k}^{i}\right)}^{T}{\left(G_{q,k}^{i}\right)}^{-1}H_{q,k}^{i}\right)|}_{\xi_{q,k|q,k-1}}$$

The data information matrix $J_{D}\left(\xi_{q,k}\right)$ is a function of radar transmit power $P_{q,k}^{i}$ and target RCS $h_{q,k}^{i}$. So BIM $J\left(\xi_{q,k}\right)$ can be rewritten as:

(22) $$\begin{array}{ll} {J\left(P_{q,k}^{i},h_{q,k}\right)|}_{\xi_{q,k}} & =J_{P}\left(\xi_{q,k}\right)+{J_{D}\left(P_{q,k}^{i},h_{q,k}\right)|}_{\xi_{q,k}}\\ & ={\left[Q_{q,k-1}+F_{q,k}J^{-1}\left(\xi_{q,k}\right)F_{q,k}{}^{T}\right]}^{-1}\\ & +{\left(\sum_{i=1}^{m}{\left(H_{q,k}^{i}\right)}^{T}{\left(G_{q,k}^{i}\left(P_{q,k}^{i},h_{q,k}^{i}\right)\right)}^{-1}H_{q,k}^{i}\right)|}_{\xi_{q,k|q,k-1}} \end{array}$$

Reverse the matrix $J\left(P_{q,k}^{i},h_{q,k}\right)$ to get the corresponding BCRLB matrix:

(23) $$C_{BCRLB}^{q}\left(P_{q,k}^{i},h_{q,k}\right)=J^{-1}\left(P_{q,k}^{i},h_{q,k}\right)$$

The $C_{BCRLB}^{q}\left(P_{q,k}^{i},h_{q,k}\right)$ is the functions of parameters such as radar transmit power. Therefore, the following formula could be used as the cost function of target allocation.

(24) $$F\left(P_{q,k}^{i},h_{q,k}\right)=\sqrt{trace\left[{C_{BCRLB}^{q}\left(P_{q,k}^{i},h_{q,k}\right)|}_{\xi_{q,k}}\right]}$$

### 4.2. Power Resource Management Opportunity Constraint Programming Model

The purpose of this section is to optimize the transmit power of each radar on the basis of meeting the preset tracking error conditions of each target. The advantage of CCP is that it can avoid some extreme constraints on the detection performance [28]. In practice, the target’s identity, attitude and orientation are uncertain. In this paper, we consider the target’s RCS as a random variable [29]. Therefore, we build the following model:

(25) $$\begin{array}{l} \min\sum_{q=1}^{N}P_{q,k}\\ s.t.\\ \begin{array}{ll} & Pr\left\{F_{q}\left(P_{q,k}^{i},h_{q,k}\right)\leq \eta_{k}\right\}\geq \alpha \\ & \sum_{i=1}^{m_{q}}P_{q,k}^{i}=P_{q,k}\\ & \sum_{q=1}^{N}m_{q}\leq M \end{array} \end{array}$$

where, α is the confidence level, which represents the probability of the random constraint being established, and is affected by many factors, such as types, attitude and mobility of the target. $m_{q}$ is the number of radars observed on the target *q* cluster, *M* is the total number of radars, and *N* is the total number of targets,$P_{q,k}$ represents the total transmitting power of all radars that have observed target *q*.$\eta_{k}$ is the specified total tracking error threshold. $P_{q,k}^{i}$ is the transmit power of radar *i* tracking the target *q*.

In previous papers, the optimization function of a joint radar node and transmitting power resource management model usually includes two variables: the radar node and the transmitting power. This model is also the same. Solving such multiple variables optimization problems usually requires a large amount of calculation. In order to avoid multiple variables in the optimization function, we designed the above FLR-based radar-clustering algorithm. So the radar node allocation problem is solved online. After radar clustering, the former CCP model can be transformed into:

(26) $$\begin{array}{l} \sum_{q=1}^{N}\min\sum_{i=1}^{m}P_{q,k}^{i}\\ s.t.\\ \begin{array}{lll} & P_{min}\leq P_{q,k}^{i}\leq P_{max} & i=1,2,\ldots ,m_{q}\\ & Pr\left\{F_{q}\left(P_{q,k}^{i},h_{q,k}\right)\leq \eta_{k}\right\}\geq \alpha & \end{array} \end{array}$$

### 4.3. Model Solving Method

Embedding stochastic simulation into the genetic algorithm to form a hybrid intelligent algorithm can solve the problem of CCP with a confidence level of α, so as to predict the transmit power $P_{q,k+1}^{i}$ of each radar in the next sampling period [28].

#### 4.3.1. Stochastic Simulation

Use Algorithm 1 to verify whether the decision vector $P_{q,k}^{i}$ meets the confidence level *a* [28].
**Algorithm 1.** Stochastic simulation algorithm**Step (1):** Let $N^{\prime }=0$.**Step (2):** Generate *N* sets of RCS vector samples ${\left[h_{k}=h_{1,k,},h_{2,k},\ldots ,h_{Q,k}\right]}_{i}\left(i=1,2,\ldots ,N\right)$ from sample space ${\left(\Omega ,A,Pr\right)}_{q}\left(q=1,2,\ldots ,Q\right)$.**Step (3):** If $\sum_{q=1}^{Q}\left[w_{q,k}\cdot F\left(P_{q,k}^{i},h_{q,k}\right)\right]\leq \eta_{k}$, then $N^{\prime }=N^{\prime }+1$. Repeat steps 2 to 3.**Step (4):** Let $Pr\left\{P_{q,k}^{i}\right\}=N^{\prime }/N$.**Step (5):** If $Pr\left\{P_{q,k}^{i}\right\}\geq \alpha$, then $P_{q,k}^{i}$ satisfies the confidence level α, otherwise it does not.

#### 4.3.2. Hybrid Intelligent Algorithm

At the *k*th sampling time, the transmit power of the radar at the next time is predicted by Algorithm 2:
**Algorithm 2.** Stochastic simulation algorithm**Step (1):** According to the target motion equation $\xi_{q,k+1|k}=F\times \xi_{q,k}$ under zero process noise, predict the state vector $\xi_{q,k+1|k}$ at the k+1 moment;.**Step (2):** Initialize population *S* and stain length *N*, then verify the feasibility of chromosomes using random simulation.**Step (3):** Hybridize and mutate chromosomes, use random simulation to verify whether the chromosomes meet the constraints, and correct those chromosomes that do not meet the constraints.**Step (4):** Calculate the objective function value of all chromosomes, and calculate the fitness function value of each stain according to the objective function value.**Step (5):** Use roulette to select chromosomes. If the requirements of the stopping rules are not met, go to (3). If the stopping rules are met, go to step (6).**Step (6):** Returns the best chromosome as the optimal radar transmit power $P_{q,k+1,opt}^{i}$.

## 5. Target State Estimation

The state vector of the target is estimated from the radar measurement information. Since measurement equations are highly nonlinear, this paper uses Algorithm 3 to predict the state vector of each target.
**Algorithm 3.** UKF algorithm**Step (1):** Let *k* = 1, initialize each radar transmit power $P_{q,k}^{i}$, target state $\xi_{q,k-1|k-1}$ and covariance matrix $C_{q,k-1|k-1}=J^{-1}\left(\xi_{q,k-1|k-1}\right)$.**Step (2):** The transmitting power of each radar is $P_{q,k}^{i}$, get the measured value $z_{q,k}$ of the target, and calculate $\Sigma_{q,k}$.**Step (3):** Construct the 2*L +* 1 sigma point set $\chi_{q,k-1|k-1}^{i}$ and the weights $\omega_{i,k}$ corresponding to the point set according to the following formula. $\{\begin{array}{ll} \chi_{q,k-1|k-1}^{0}=\xi_{q,k-1|k-1} & \\ \chi_{q,k-1|k-1}^{i}=\xi_{q,k-1|k-1}+{\left(\sqrt{\left(I+\varsigma \right)C_{q,k-1|k-1}}\right)}_{i} & i=1,2,\ldots ,I\\ \chi_{q,k-1|k-1}^{i}=\xi_{q,k-1|k-1}-{\left(\sqrt{\left(I+\varsigma \right)C_{q,k-1|k-1}}\right)}_{i-I} & i=I+1,I+2,\ldots ,2I \end{array}$
$\{\begin{array}{ll} \omega_{0,k}=\frac{\varsigma }{I+\varsigma } & \\ \omega_{i,k}=\frac{I}{2\left(I+\varsigma \right)} & i=1,2,\ldots ,2I \end{array}$
Where, ς is a scale factor, ${\left(\sqrt{\left(I+\varsigma \right)C_{q,k-1|k-1}}\right)}_{i}$ represents the *i*th column of the square root $\sqrt{\left(I+\varsigma \right)C_{q,k-1|k-1}}$ of the matrix; *I* represents the dimension of the state vector.**Step (4):** Map the sigma point set $\chi_{q,k-1|k-1}^{i}$ to the predicted point set $\chi_{q,k|k-1}^{i}$ through the state transition function $F_{q}$, and calculate the new target state $\xi_{q,k|k-1}$ and variance $C_{q,k|k-1}$ by weighting.
$\chi_{q,k|k-1}^{i}=F_{q}\times \chi_{q,k-1|k-1}^{i}$ $\{\begin{array}{l} \xi_{q,k|k-1}=\sum_{i=0}^{2I}\left(\omega_{i,k}\times \chi_{q,k|k-1}^{i}\right)\\ C_{q,k|k-1}=\sum_{i=0}^{2I}\omega_{i,k}\left(\chi_{q,k|k-1}^{i}-\xi_{q,k|k-1}\right){\left(\chi_{q,k|k-1}^{i}-\xi_{q,k|k-1}\right)}^{T}+Q_{q,k-1} \end{array}$**Step (5):** Map the sigma prediction point set $\chi_{q,k|k-1}^{i}$ to the new point set $z_{q,k|k-1}^{i}$ through the measurement equation, and calculate the mean $z_{q,k|k-1}$, variance $C_{q,k}^{zz}$ and $C_{q,k}^{xz}$. $z_{q,k|k-1}^{i}=g\left(\chi_{q,k|k-1}^{i}\right)$ $\{\begin{array}{l} z_{q,k|k-1}=\sum_{i=0}^{2I}\left(\omega_{i,k}\times z_{q,k|k-1}^{i}\right)\\ C_{q,k}^{zz}=\sum_{i=0}^{2I}\omega_{i,k}\left(z_{q,k|k-1}^{i}-z_{q,k|k-1}\right){\left(z_{q,k|k-1}^{i}-z_{q,k|k-1}\right)}^{T}+\Sigma_{q,k}\\ C_{q,k}^{zz}=\sum_{i=0}^{2I}\omega_{i,k}\left(\chi_{q,k|k-1}^{i}-\xi_{q,k|k-1}\right){\left(\chi_{q,k|k-1}^{i}-\xi_{q,k|k-1}\right)}^{T} \end{array}$**Step (6):** Calculate the gain matrix $K_{q,k}$ and update $\xi_{q,k|k-1}$ and covariance $C_{q,k|k}$ matrix with the gain matrix. $\{\begin{array}{l} K_{q,k}=C_{q,k}^{xz}\times {\left(C_{q,k}^{zz}\right)}^{-1}\\ \xi_{q,k|k}=\xi_{q,k|k-1}+K_{q,k}\left(z_{q,k}-z_{q,k|k-1}\right)\\ C_{q,k|k}=C_{q,k|k-1}-K_{q,k}\times C_{q,k}^{zz}\times {\left(K_{q,k}\right)}^{T} \end{array}$
**Step (7):** According to the intelligent hybrid algorithm proposed above, predict the radar transmission power $p_{k+1,opt}^{i}$ at the *k*th moment, let k=k+1 and then jump to step (2)

## 6. Simulation Results and Analysis

This section designs a scenario where multiple radars are tracking multiple targets to verify the effectiveness of the above algorithm. In order to simplify the analysis and eliminate the influence of radar deployment on the tracking performance, the positions of the 12 radars in the scene are not changed, and all radars form a square network. There are three targets in the scene, and the starting position of each target is set randomly. The radar parameters of each radar are the same, as shown in the following Table 2.

**Figure 6:**
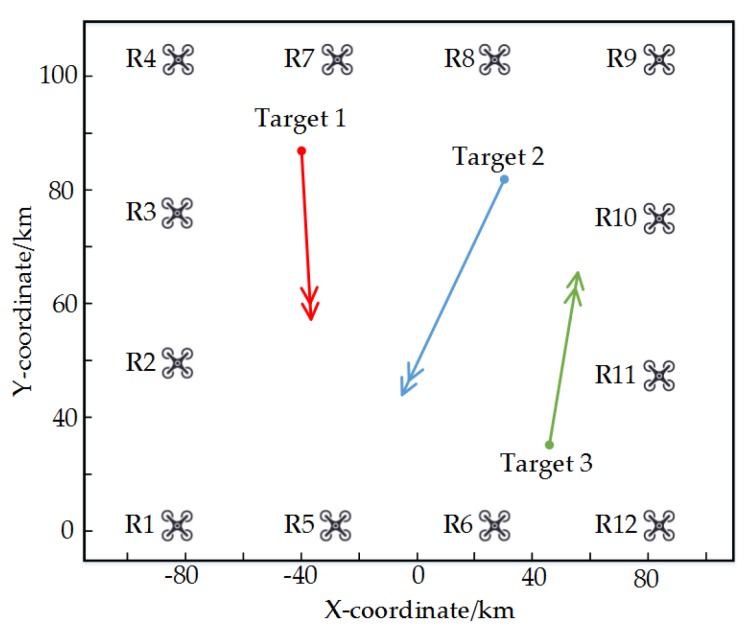
The distribution of radars and targets.

The position parameters of radars are shown in Table 3:

The status parameters of the three targets are shown in the Table 4:

The distribution of radar and targets is shown in Figure 6:

There are 40 frames in the simulation process. Within each frame, the radar clustering method based on FLR is first used to implement the selection of radar node. Then, the CCP model is used to allocate the transmitting power of each radar.

**Figure 7:**
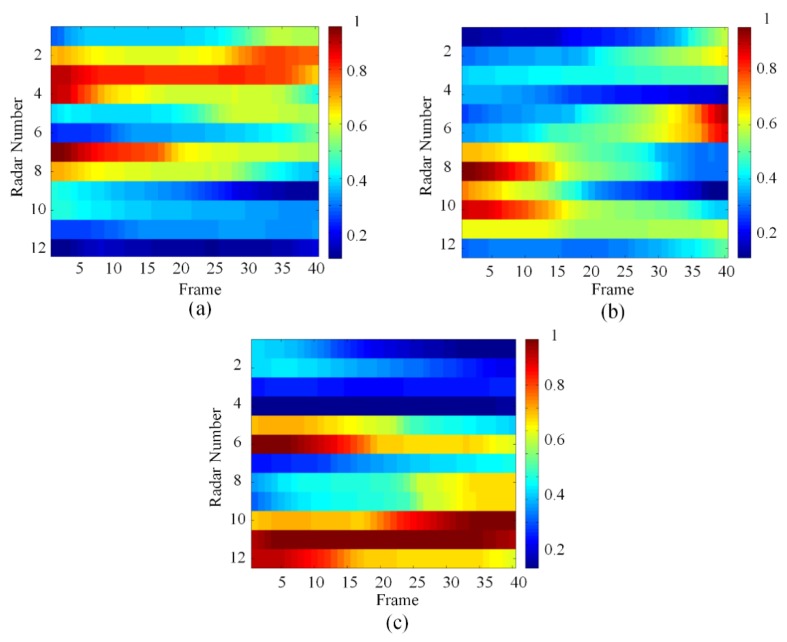
The priorities of each target: (**a**) target 1, (**b**) target 2, and (**c**) target 3.

In order to facilitate comparison, this paper extracts all the numerical results $w^{*}$ obtained by FLR at each moment and displays them uniformly. So the simulation results of the priority of each target to different radars are shown below:

Figure 7 shows all the numerical solutions $w^{*}$ obtained by FLR, which can show the change of the priority of the targets for each radar. The value of $w^{*}$ is between (0,1). The legend on the right shows the correspondence between colors and numerical values of $w^{*}$. Warm colors represent higher numerical value $w^{*}$, which means the target has a higher priority, while cool tones have the opposite. As the picture shows, target 1 has high priority for radars 2, 3, 4, and 7 at the beginning. As the target 1 moves, its priority for radars 4 and 7 gradually decreases, and for radar 2 it gradually increases. The priority change of target 2 is similar to target 1, first, the priority of target 1 two for radars 8, 9, and 10 is high. As it moves, its priority for radars 5 and 6 gradually increases. The situation of target 3 is slightly different. Because target 3 is closer to radar 11, its priority for radar 11 is always high. So, it can be proved by the simulation results that as the target moves, the targets’ priorities for different radars will change accordingly.

According to the above numerical values in Figure 7, using the radar node selection algorithm proposed in Section 3.2, the clustering situation of radar and target at each moment can be obtained.

**Figure 8:**
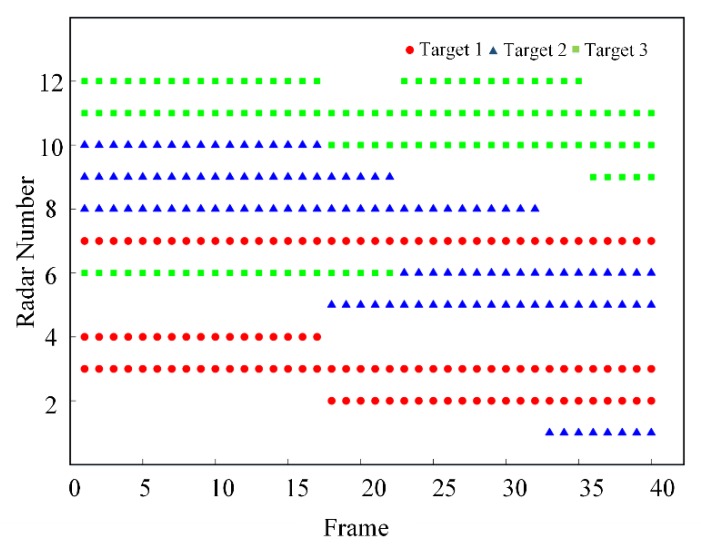
Radar clustering at each moment.

Figure 8 shows the radar-clustering situation for each target at different time. It can be seen from the figure that each target is always tracked by three radars from different angles. Combining Figure 6 and Figure 8, it can be found that only three radars that are relatively close to the target will cluster and track it at the same time. The number of radars involved in tracking target 2 is the largest during the entire tracking process. This is because target 2 has the fastest speed and the widest area of motion coverage.

After obtaining the clustering situation of radars for targets at each moment. The hybrid intelligent algorithm is used to solve the power CCP model for multiple-radar tracking single-target. From this, the total transmitting power of each radar can be obtained.

**Figure 9:**
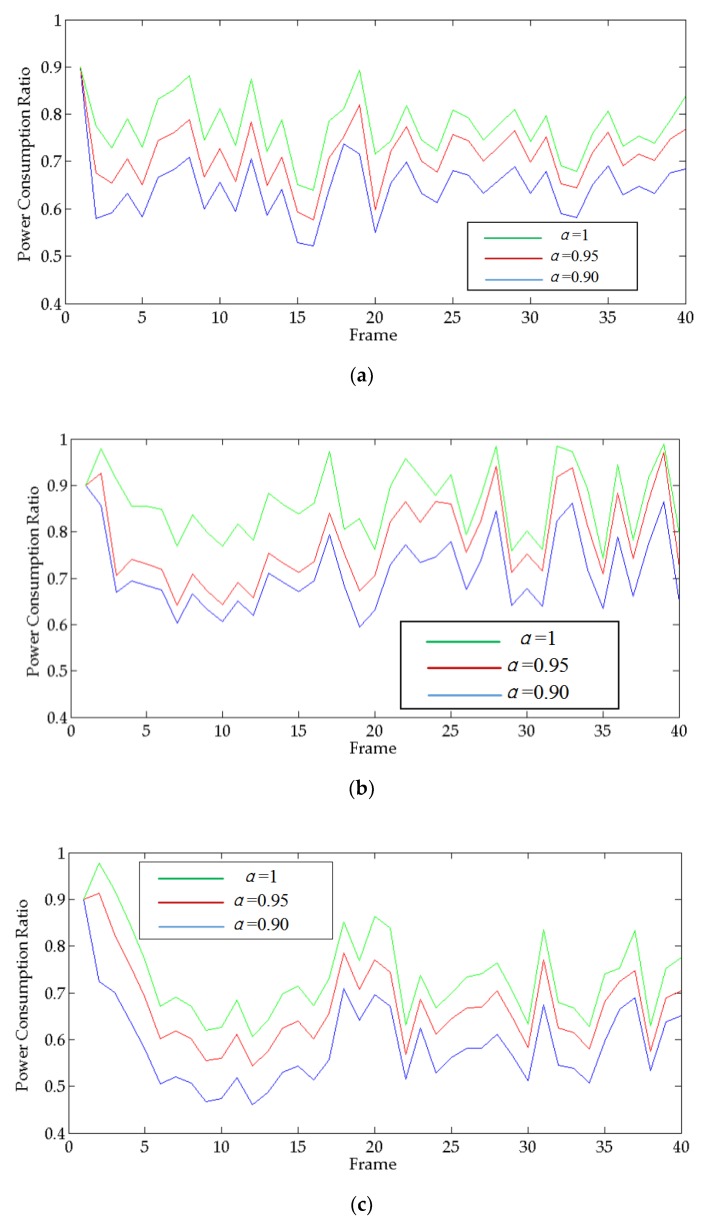
Total power consumption ratio: (**a**) Target 1; (**b**)Target 1; (**c**)Target 1.

Figure 9 shows the total power of the radars tracking each target at each moment. Where α is the confidence level. It can be found that as the confidence level α decreases, the transmit power also decreases. When α is reduced by 0.5, the radar can save nearly 9% of the total power. In this article, RCS is set as a random variable, so the transmission power fluctuates randomly following the change of RCS. Comparing the total transmit power of the three targets, it can be found that the total transmit power of target 2 is the highest, and the total transmit power of target 3 is the lowest, which is caused by the distance between the target and the radar. The above simulation results prove that this algorithm can save large transmit power.

**Figure 10:**
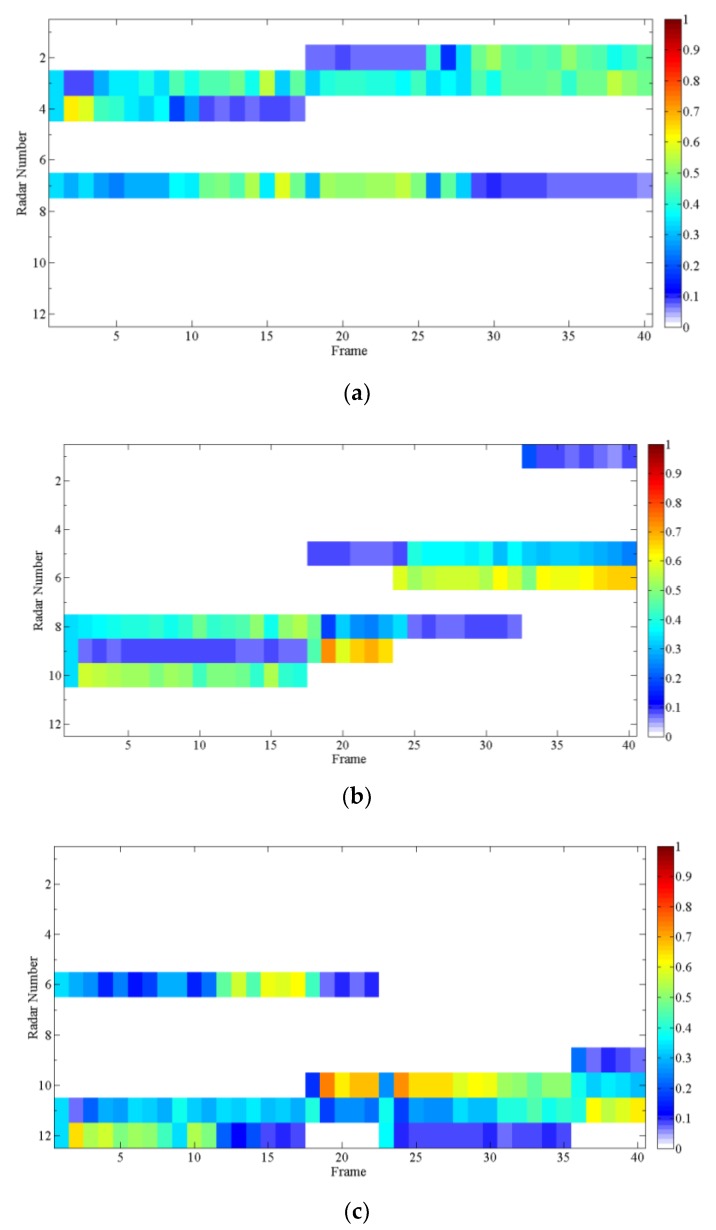
Percentage of radar transmitting power: (**a**) Target 1; (**b**) Target 2; (**c**) Target 3.

In order to clearly show the distribution of the transmit power for each radar, Figure 10 gives the ratio of the transmitting power of each radar to the total transmitting power when the confidence level α is 0.9.

It is found from the three diagrams in Figure 10 that each target is always tracked by three radars, and it is very rare that one radar operates at extremely high power. It proves that the algorithm effectively limits the transmitting power of each radar. The transmitting power of each radar in Figure 10 is in different forms of fluctuations, because each radar observes the target at different angles. 

**Figure 11:**
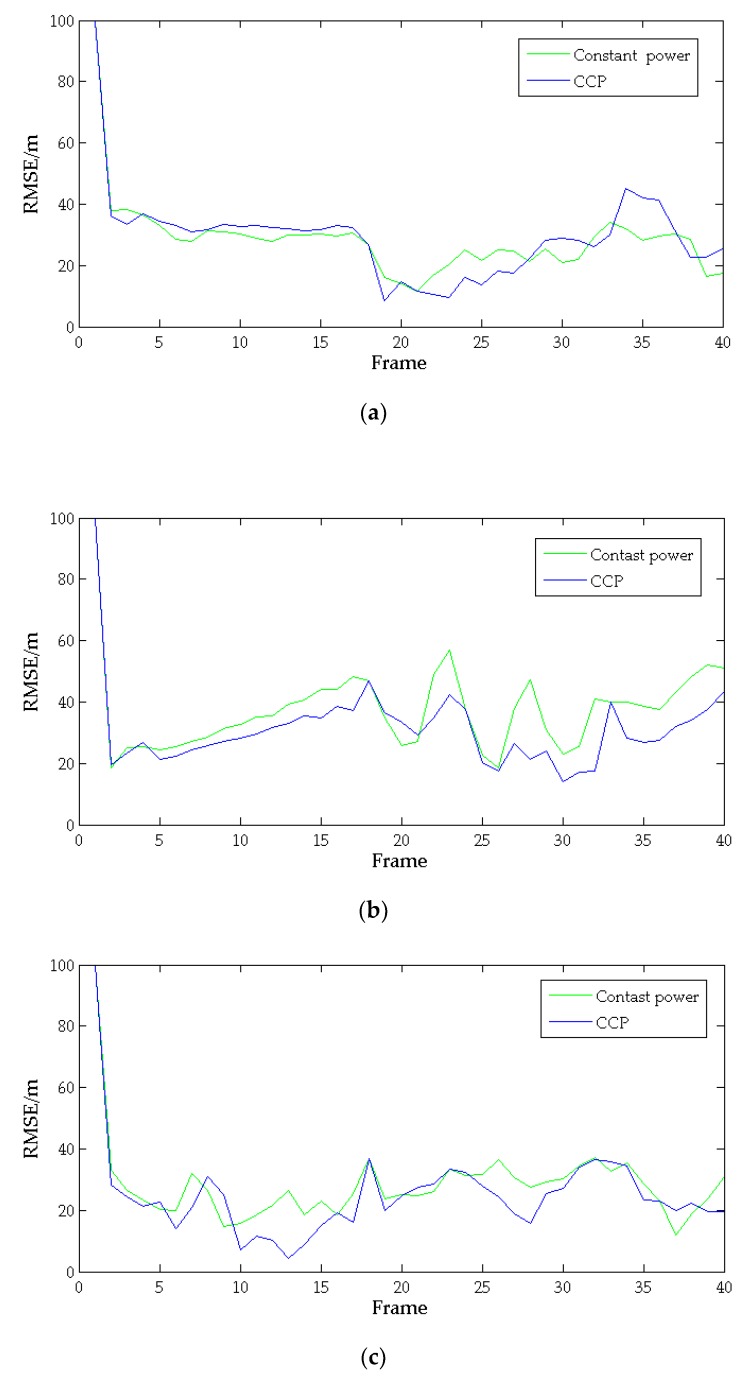
The RMSE of each target tracking error: (**a**) Target 1; (**b**) Target 2; (**c**) Target 3.

In order to show the tracking performance of the algorithm in this paper, we compare the root mean square error(RMSE) of target tracking error obtained by this algorithm with the RMSE of equal power allocation in Figure 11.

Figure 11 shows the changes in the RMSE of each target tracking error at different times. When α is 0.9, the RMSE obtained by the CCP model is represented by the blue curve. When each radar uses 90% of its own power to track the targets, the RMSE of each target is represented by the green curve. Because of the randomness of the target RCS, the RMSE of both also fluctuate. It can be seen from the Figure 11 that the gap between the blue and green lines of each target is acceptable, which can prove that the algorithm in this paper is in high tracking accuracy while effectively saving power.

## 7. Conclusions

This paper provides a new solution to the issues of joint radar node selection and power allocation for radar networks based on UAV. An innovative radar-clustering algorithm is proposed to select the radar node online. In the paper, the target RCS is treated as a random variable to ensure the stability of the model in real scenarios. Aiming at the problem that the size of the UAV limits the radar transmitting power, the CCP model is used to optimize radar transmit power. Simulation results show that the algorithm in this paper can reasonably schedule radar node and effectively save the radar power resources.

The future work will be directed to the following aspects: The paper assumes that the position of each drone is fixed. This does not fully take advantage of unmanned cluster. In the future, we can rationally schedule the layout of UAVs based on the target-tracking situation to achieve better tracking performance.

## Figures and Tables

**Table 1:** Fuzzy values related to fuzzy variables.

Fuzzy Variable	Excursion	Fuzzy Value
range	(0,360] (km)	close, medium-close, medium, medium-far, far
speed	(0,960] (m/s)	very slow, slow, medium, fast, very fast
target identity	(0,1]	low risk, medium risk, high risk
priority	(0,1]	low, medium, high

**Table 2:** Parameters of radars.

Parameter	Wavelength	Effective Bandwidth	Effective Timewidth	Sampling Interval
Value	0.03 m	5 MHz	1 ms	0.5 s

**Table 3:** Position parameters of radars.

Radar Serial Number	Position/(m, m)
1	(−82,500, 0)
2	(−82,500, 55,000)
3	(−82,500, 110,000)
4	(−82,500, 165,000)
5	(−27,500, 0)
6	(27,500, 0)
7	(−27,500, 165,000)
8	(27,500, 165,000)
9	(82,500, 165,000)
10	(82,500, 110,000)
11	(82,500, 55,000)
12	(82,500, 0)

**Table 4:** State parameter of targets.

Target	Initial position/(m, m)	Velocity/(m/s)
1	(−40,000, 135,000)	(30, −520)
2	(31,000, 125,000)	(−310, −660)
3	(45,500, 29,000)	(90, 530)

## Appendix A

Based on expert experience and experimental data, 63 if-then rules are listed below:

1. IF (range is close)  AND (speed is very slow) AND (identity is low risk), then (priority is Medium)
2. IF (range is close)  AND (speed is very slow) AND (identity is medium risk), then (priority is High)
3. IF (range is close) AND (speed is very slow) AND (identity is high risk), then (priority is High)
4. IF (range is close) AND (speed is slow) AND (identity is low risk), then (priority is Medium)
5. IF (range is close) AND (speed is slow) AND (identity is medium risk), then (priority is High)
6. IF (range is close) AND (speed is slow) AND (identity is high risk), then (priority is High)
7. IF (range is close) AND (speed is medium) AND (identity is low risk), then (priority is Medium)
8. IF (range is close) AND (speed is medium) AND (identity is medium risk), then (priority is High)
9. IF (range is close) AND (speed is medium) AND (identity is high risk), then (priority is High)
10. IF (range is close) AND (speed is fast) AND (identity is low risk), then (priority is High)
11. IF (range is close) AND (speed is fast) AND (identity is medium risk), then (priority is High)
12. IF (range is close) AND (speed is fast) AND (identity is high risk), then (priority is High)
13. IF (range is close) AND (speed is very fast) AND (identity is low risk), then (priority is High)
14. IF (range is close) AND (speed is very fast) AND (identity is medium risk), then (priority is High)
15. IF (range is close) AND (speed is very fast) AND (identity is high risk), then (priority is High)
16. IF (range is medium-close) AND (speed is very slow) AND (identity is low risk), then (priority is Medium)
17. IF (range is medium-close) AND (speed is very slow) AND (identity is medium risk), then (priority is Medium)
18. IF (range is medium-close) AND (speed is very slow) AND (identity is high risk), then (priority is Medium)
19. IF (range is medium-close) AND (speed is slow) AND (identity is low risk), then (priority is Medium)
20. IF (range is medium-close) AND (speed is slow) AND (identity is medium risk), then (priority is Medium)
21. IF (range is medium-close) AND (speed is slow) AND (identity is high risk), then (priority is High)
22. IF (range is medium-close) AND (speed is medium) AND (identity is low risk), then (priority is Medium)
23. IF (range is medium-close) AND (speed is medium) AND (identity is medium risk), then (priority is Medium)
24. IF (range is medium-close) AND (speed is medium) AND (identity is high risk), then (priority is High)
25. IF (range is medium-close) AND (speed is fast) AND (identity is low risk), then (priority is Medium)
26. IF (range is medium-close) AND (speed is fast) AND (identity is medium risk), then (priority is High)
27. IF (range is medium-close) AND (speed is fast) AND (identity is high risk), then (priority is High)
28. IF (range is medium-close) AND (speed is very fast) AND (identity is low risk), then (priority is Medium)
29. IF (range is medium-close) AND (speed is very fast) AND (identity is medium risk), then (priority is High)
30. IF (range is medium-close) AND (speed is very fast) AND (identity is high risk), then (priority is High)
31. IF (range is medium) AND (speed is very slow) AND (identity is low risk), then (priority is Low)
32. IF (range is medium) AND (speed is very slow) AND (identity is medium risk), then (priority is Medium)
33. IF (range is medium) AND (speed is very slow) AND (identity is high risk), then (priority is Medium)
34. IF (range is medium) AND (speed is slow) AND (identity is low risk), then (priority is Low)
35. IF (range is medium) AND (speed is slow) AND (identity is medium risk), then (priority is Medium)
36. IF (range is medium) AND (speed is slow) AND (identity is high risk), then (priority is Medium)
37. IF (range is medium) AND (speed is medium) AND (identity is low risk), then (priority is Medium)
38. IF (range is medium) AND (speed is medium) AND (identity is medium risk), then (priority is Medium)
39. IF (range is medium) AND (speed is medium) AND (identity is high risk), then (priority is Medium)
40. IF (range is medium) AND (speed is fast) AND (identity is low risk), then (priority is Medium)
41. IF (range is medium) AND (speed is fast) AND (identity is medium risk), then (priority is Medium)
42. IF (range is medium) AND (speed is fast) AND (identity is high risk), then (priority is High)
43. IF (range is medium) AND (speed is very fast) AND (identity is low risk), then (priority is Medium)
44. IF (range is medium) AND (speed is very fast) AND (identity is medium risk), then (priority is Medium)
45. IF (range is medium) AND (speed is very fast) AND (identity is high risk), then (priority is High)
46. IF (range is medium-far) AND (speed is very slow) AND (identity is low risk), then (priority is Low)
47. IF (range is medium-far) AND (speed is very slow) AND (identity is medium risk), then (priority is Low)
48. IF (range is medium-far) AND (speed is very slow) AND (identity is high risk), then (priority is Medium)
49. IF (range is medium-far) AND (speed is slow) AND (identity is low risk), then (priority is Low)
50. IF (range is medium-far) AND (speed is slow) AND (identity is medium risk), then (priority is Low)
51. IF (range is medium-far) AND (speed is slow) AND (identity is high risk), then (priority is Medium)
52. IF (range is medium-far) AND (speed is medium) AND (identity is low risk), then (priority is Low)
53. IF (range is medium-far) AND (speed is medium) AND (identity is medium risk), then (priority is Low)
54. IF (range is medium-far) AND (speed is medium) AND (identity is high risk), then (priority is Medium)
55. IF (range is medium-far) AND (speed is fast) AND (identity is low risk), then (priority is Low)
56. IF (range is medium-far) AND (speed is fast) AND (identity is medium risk), then (priority is Medium)
57. IF (range is medium-far) AND (speed is fast) AND (identity is high risk), then (priority is Medium)
58. IF (range is medium-far) AND (speed is very fast) AND (identity is low risk), then (priority is Low)
59. IF (range is medium-far) AND (speed is very fast) AND (identity is medium risk), then (priority is Medium)
60. IF (range is medium-far) AND (speed is very fast) AND (identity is high risk), then (priority is Medium)
61. IF (range is far) AND (speed is very slow) AND (identity is low risk), then (priority is Low)
62. IF (range is far) AND (speed is very slow) AND (identity is medium risk), then (priority is Low)
63. IF (range is far) AND (speed is very slow) AND (identity is high risk), then (priority is Medium)
64. IF (range is far) AND (speed is slow) AND (identity is low risk), then (priority is Low)
65. IF (range is far) AND (speed is slow) AND (identity is medium risk), then (priority is Low)
66. IF (range is far) AND (speed is slow) AND (identity is high risk), then (priority is Medium)
67. IF (range is far) AND (speed is medium) AND (identity is low risk), then (priority is Low)
68. IF (range is far) AND (speed is medium) AND (identity is medium risk), then (priority is Low)
69. IF (range is far) AND (speed is medium) AND (identity is high risk), then (priority is Medium)
70. IF (range is far) AND (speed is fast) AND (identity is low risk) then (priority is Low)
71. IF (range is far) AND (speed is fast) AND (identity is medium risk), then (priority is Low)
72. IF (range is far) AND (speed is fast) AND (identity is high risk), then (priority is Medium)
73. IF (range is far) AND (speed is very fast) AND (identity is low risk), then (priority is Low)
74. IF (range is far) AND (speed is very fast) AND (identity is medium risk), then (priority is Low)
75. IF (range is far) AND (speed is very fast) AND (identity is high risk), then (priority is Medium)
